# A dose‐volume‐based tool for evaluating and ranking IMRT treatment plans

**DOI:** 10.1120/jacmp.v5i4.1981

**Published:** 2004-11-24

**Authors:** Moyed M. Miften, Shiva K. Das, Min Su, Lawrence B. Marks

**Affiliations:** ^1^ Department of Radiation Oncology Duke University Medical Center Durham North Carolina 27710 U.S.A.

**Keywords:** treatment planning, IMRT, plan evaluation, dose volume

## Abstract

External beam radiotherapy is commonly used for patients with cancer. While tumor shrinkage and palliation are frequently achieved, local control and cure remain elusive for many cancers. With regard to local control, the fundamental problem is that radiotherapy‐induced normal tissue injury limits the dose that can be delivered to the tumor. While intensity‐modulated radiation therapy (IMRT) allows for the delivery of higher tumor doses and the sparing of proximal critical structures, multiple competing plans can be generated based on dosimetric and/or biological constraints that need to be considered/compared. In this work, an IMRT treatment plan evaluation and ranking tool, based on dosimetric criteria, is presented. The treatment plan with the highest uncomplicated target conformity index $\left({\text{TCI}}^{+}\right)$ is ranked at the top. The ${\text{TCI}}^{+}$ is a dose‐volume‐based index that considers both a target conformity index (TCI) and a normal tissue‐sparing index (NTSI). ${\text{TCI}}^{+}$ is designed to assist in the process of judging the merit of a clinical treatment plan. To demonstrate the utility of this tool, several competing lung and prostate IMRT treatment plans are compared. Results show that the plan with the highest ${\text{TCI}}^{+}$ values accomplished the competing goals of tumor coverage and critical structures sparing best, among rival treatment plans for both treatment sites. The study demonstrates, first, that dose‐volume‐based indices, which summarize complex dose distributions through a single index, can be used to automatically select the optimal plan among competing plans, and second, that this dose‐volume‐based index may be appropriate for ranking IMRT dose distributions.

PACS numbers: 87.53.‐j, 87.53.Tf

## I. INTRODUCTION

Intensity‐modulated radiation therapy (IMRT) is currently implemented in many academic and community clinics. Basic and clinical research work shows that IMRT dose distributions are highly conformal and complex.^(^ ^1^ ^–^ ^5^ ^)^ While the power of IMRT is to conform the high‐dose volume to the target and spare adjacent critical structures, dose distributions of IMRT plans are typically much more heterogeneous than those of conventional 3D‐derived plans.^(^ ^6^ ^,^ ^7^ ^)^ Comparing competing IMRT plans becomes a challenging process. The very complex nature of IMRT‐derived plans challenges the application of traditional figures of merit (e.g., mean and minimum dose) to assess plans. Furthermore, the increased flexibility afforded by IMRT increases the number of possible plans beyond the already high number of possible plans based on traditional 3D planning. For example, a number of IMRT treatment plans, with different dosimetric outcomes, can be generated based on dose‐volume or dose‐response constraints.^(^ ^8^ ^–^ ^10^ ^)^

Equivalent uniform dose,^(^ ^11^ ^)^ dose‐volume reduction schemes,^(^ ^12^ ^)^ and objective functions in inverse planning algorithms are dose‐volume‐based indices that have been used to evaluate external beam plans. Furthermore, several volume‐based dosimetric indices have been proposed to numerically quantify the quality of dose distributions. The indices have generally been applied to brachytherapy dose distributions and stereotactic dose distributions/^13‐15^ Knöös et al. reported a target conformity index to evaluate conformal treatment plans based on target dose‐volume definitions of ICRU 50.^(^ ^16^ ^,^ ^17^ ^)^ The index, however, did not quantify the irradiated critical structure volumes surrounding the target.

The selection of an “optimal plan” from among competing IMRT treatment plans for cancer therapy is a daunting task. Currently, planners base plan evaluation/selection on visual inspection of the isodose distributions and dose‐volume histograms. A plan is deemed satisfactory if certain normal tissue dose criteria are met and the isodose lines indicate “good” target coverage. However, this selection technique can be ambiguous, since, in many cases, one can generate multiple plans using different objective functions that may be deemed satisfactory. For these reasons, traditional methods of evaluating and ranking treatment plans may be too limited for IMRT dose distributions. This creates the need for an independent dose‐volume index to rank IMRT treatment plans that are generated using either different objective functions or the same objective function but using different dose‐volume constraints (or different importance parameters).

In this work, an automated IMRT treatment plan evaluation and ranking tool based on physical indices is developed and herein presented. The ranking algorithm selects the plan that maximizes an uncomplicated target conformity index $\left({\text{TCI}}^{+}\right)$. The ${\text{TCI}}^{+}$ is a dose‐volume‐based index that combines the objectives of maximizing the conformation of a therapeutic dose to the target and minimizing exposure to normal tissues. In essence, this index quantifies clinician judgment of the merit of a treatment plan, and is formulated by conferring with physicians at our institution during the course of treatment‐planning sessions. To demonstrate the utility of the ${\text{TCI}}^{+}$ plan ranking tool, competing IMRT treatment plans for patients with lung and prostate cancers are compared using the ${\text{TCI}}^{+}$ formulation.

## II. METHODS

**Figure 1:**
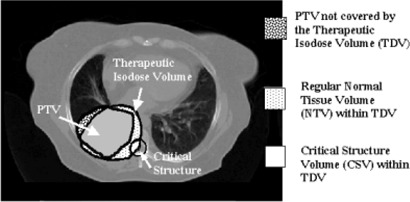
Illustration of volumes of interest for target, regular normal tissue, and critical structures enclosed by a therapeutic isodose volume on a lung CT slice. The volumes are used to calculate a target conformity index (TCI) and normal‐tissue sparing index (NTSI). The a “speculative” situation, where a fraction of the target is not covered by the therapeutic isodose volume (TDV), and a fraction of the spinal cord volume is within TDV.

The ${\text{TCI}}^{+}$ consists of two components: the target conformity index (TCI) and the normal tissue‐sparing index (NTSI), explained below. Figure 1 illustrates the different dose‐volume quantities used to calculate TCI and NTSI on a lung computed tomography (CT) transverse slice. The normal tissue is differentiated as “regular” normal tissue and “critical structure” normal tissue. Any tissue in the treated volume that is not contoured and not part of the target volume or critical structure volumes is considered regular normal tissue volume.

### A. Target conformity index

The TCI is a figure of merit calculated from the target dose‐volume data as

(1) $$\text{TCI}=P_{\text{PTV}}\left(\frac{{\text{PTV}}_{\text{TD}}}{\text{PTV}}\right)$$

The bracketed term of the TCI equation reports the fraction of the planning target volume (PTV) that receives a minimum specified therapeutic dose (TD).^(^ ^13^ ^,^ ^16^ ^)^ ${\text{PTV}}_{\text{TD}}$ is the part of the PTV enclosed by the therapeutic dose (i.e., the therapeutic isodose volume, TDV). $P_{\text{PTV}}$ a target penalty function that uses dosimetric metrics to penalize over/underdosage of target subvolumes.

The penalty function specifies limits for subvolumes of the target that are permitted to receive dose above/below specified maximum/minimum dose limits and the penalty values associated with their violation. The penalty values range between 0 and 1. A value of 1 indicates no penalty is enforced, and a value of less than 1 implies a penalty is imposed based on target dose‐volume violations, thus decreasing the TCI value due to a hot or cold spot. A range of penalty values may be defined based on the treatment site and clinical experience.

Target conformity index reports target dose coverage as a value between 0 and 1. A value of 1 indicates an ideal plan with target coverage by TDV with no over/underdosage of target subvolumes (i.e., $P_{\text{PTV}}=1$); a TCI value of 0 indicates the whole target volume is not covered by TDV (i.e., ${\text{PTV}}_{\text{TD}}=0)$ or the existence of a severe cold spot(s) in the target which resulted in a penalty of zero.

**Figure 2:**
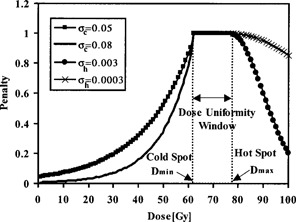
A target penalty function used in the calculation of TCI with different values for $\sigma_{c}$ and $\sigma_{h}$. The penalty function does not penalize the plan if the target dose lies in the uniformity window. If the target dose is higher than a maximum dose or lower than a minimum dose, a penalty will be enforced based on the magnitude of dose‐volume violation and the type of violation (hot spot or cold spot).

From the target differential dose‐volume histogram data, we calculate the penalty values associated with a user‐defined dose‐volume violations for a subvolume, *i,* of the target receiving a dose lower than an allowable minimum or higher than an allowable maximum dose as

(2) $$P_{\text{PTV}}(V_{i},D_{i})=\{\begin{array}{ll} e^{‐\sigma_{c,i}(D_{\text{min}}‐D_{i})} & \text{for}\;V_{i}>V_{c,\text{max}}\;\text{and}\;D_{i}<D_{\text{min}}\\ 1 & \text{for}\;D_{\text{min}}\leq D_{i}\leq D_{\text{max}}\\ e^{‐\sigma_{h,i}{\left(D_{i}‐D_{\text{max}}\right)}^{2}} & \text{for}\;V_{i}>V_{h,\text{max}}\;\text{and}\;D_{i}>D_{\text{max}} \end{array}$$

where $D_{\text{min}}$ and $D_{\text{max}}$ are the PTV minimum and maximum allowed doses, respectively $V_{c,\max}$ is the maximum allowable fractional volume of the target receiving dose below $D_{\text{min}}$ (i.e., cold spot in a target subvolume). $V_{h,\max}$ is the maximum allowable fractional volume of the target receiving dose above $D_{\text{max}}$ (i.e., hot spot in a target subvolume). The subscripts “h” and “c” denote hot and cold. The penalty function for the target is shown in Fig. 2. Cold spots are drastically penalized using an exponential function, since underdosage can result in significant loss of local control, and also because target response to dose generally follows a sigmoidal distribution.^(^ ^18^ ^)^ A less drastic Gaussian function is used to penalize hot spots since it is characterized by a “shoulder” following $D_{\text{max}}$. This is consistent with clinical experience indicating that hot spots in the target are generally acceptable, provided they do not exceed a certain dose limit.

The penalty function will not penalize the plan if the target dose lies in a dose uniformity window as shown, for example, in Fig. 2 for a therapeutic dose of 70 Gy. The window is bounded by a user‐defined minimum dose limit of 62 Gy and a maximum dose limit of 76 Gy (i.e., 10% dose uniformity window). The $\sigma_{ci}$ and $\sigma_{hi}$ parameters define how rapidly the exponential and Gaussian functions decrease, thus resulting in different penalty values based on the target type, magnitude of dose‐volume violation, and the type of violation (cold or hot spot). Since tumor control declines rapidly even for a small underdosed volume, and depends strongly on the magnitude of underdosage,^(^ ^19^ ^)^ values of $\sigma_{ci}$ and $\sigma_{hi}$ can be assigned to give a higher penalty for cold spots and a lower penalty for hot spots. For example, a value of 0.08 for $\sigma_{c}$ can be used to penalize the plan for a cold spot associated with a specified target subvolume, and a value of 0.0003 for $\sigma_{h}$ can be used to penalize a hot spot associated with a specified target subvolume, as shown in Fig. 2. We used the maximum penalty values in Eq. (1) associated with the worst violation for the target:

(3) $$P_{\text{PTV}}=\underset{i}{\text{min}}[P_{\text{PTV}}(V_{i},D_{i})]$$

### B. Normal tissue‐sparing index

The NTSI considers the sparing of critical structure normal tissue and regular normal tissue volumes from high dose. It reports information on normal tissue subvolumes spared from the therapeutic isodose volume (TDV) as

(4) $$\text{NTSI}=P_{\text{NTV}}\left(1‐\frac{{\text{NTV}}_{\text{TD}}}{\text{NTV}}\right)$$

The ${\text{NTV}}_{\text{TD}}/\text{NTV}$ ratio in Eq. (4) quantifies the undesirable dose delivered to the normal tissue and reports the fraction within the therapeutic isodose volume. The normal tissue volume adjacent to the target irradiated to a high dose, which is frequently encountered in IMRT, is quantified and accounted for in the calculation of NTSI. This is depicted in Fig. 1 (a “speculative” situation where a fraction of the spinal cord volume is within TDV). The NTSI has a value between 0 and 1. A value of 1 indicates an ideal plan, where the normal tissue volume is not within TDV and thus indicates complete volume sparing of the normal tissue. A value of 0 indicates the worst plan, where the normal tissue whole volume is within TDV. Further, this implies that the whole normal tissue volume is irradiated to an undesirable high dose. NTSI can be evaluated for each critical structure and the regular normal tissue in the treated volume. To account for organ motion and setup errors, the user can consider the planning risk volume (critical structure volume plus a margin) in the NTSI calculation.^(^ ^20^ ^)^

**Figure 3:**
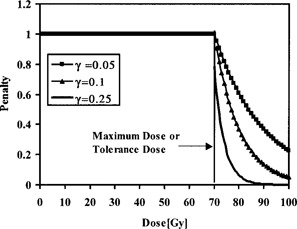
A regular normal tissue or critical structure penalty function with different γ values. The penalty function does not penalize the plan if the normal tissue dose is below a certain specified dose. If the dose is higher than the specified dose (maximum dose for regular normal tissue or tolerance dose for critical structure normal tissue), a penalty will be enforced based on the magnitude of dose‐volume violations and the type of organ (serial or parallel) for the case of critical structures.

$P_{\text{NTV}}$ is a penalty function that depends on normal tissue subvolumes exceeding tolerance doses. The penalty function $P_{\text{NTV}}$ quantifies dose‐volume violations for each critical structure using the differential dose‐volume histogram data. Thus, the risk of complications associated with such a violation is quantified. Specifically, $P_{\text{NTV}}$ specifies subvolume limits for each critical structure, which may receive dose above a tolerance dose limit (or above a maximum allowable dose for the case of regular normal tissue), and the penalty values associated with their violations. The penalty function for the normal tissue is illustrated in Fig. 3 for a tolerance dose of 70 Gy. If a normal tissue subvolume dose is equal to or below a specified tolerance dose, the penalty function will not penalize the plan. However, if the dose is above the tolerance dose, then the penalty associated with this violation is calculated as follows:

(5) $$P_{\text{NTV}}(V_{i},D_{i})=\{\begin{array}{ll} 1 & \text{for}\;D_{i}\leq D_{\text{tol}}\\ e^{‐\gamma_{i}(D_{i}‐D_{\text{tol}})} & \text{for}\;V_{i}>V_{\text{max}}\;\text{and}\;D_{i}>D_{\text{tol}} \end{array}$$

where $D_{\text{tol}}$ is the dose tolerated by a maximum fractional volume $V_{\text{max}}$ of the normal tissue. The penalty values range between 0 and 1. A value of 1 implies no penalty is enforced, and a value of less than 1 implies a penalized plan. The maximum tolerance dose and dose‐volume violation, which may cause complications, can be defined for each critical structure based on recent clinical data^(^ ^21^ ^–^ ^30^ ^)^ or other data.^(^ ^31^ ^)^ The $\gamma_{i}$ are parameters that define how rapidly the exponential function decreases, thus resulting in different penalty values based on the dose‐volume tolerance and the type of critical structure (i.e., serial or parallel organ). For example, a value of 0.25 can be used for a serial organ, compared with a value of 0.05 for a parallel organ as shown in Fig. 3. This will result in a much more severe penalty value if a plan exceeds the serial organ tolerance dose for any subvolume compared with a plan that exceeds the parallel organ tolerance dose for a similar subvolume.

**Figure 4:**
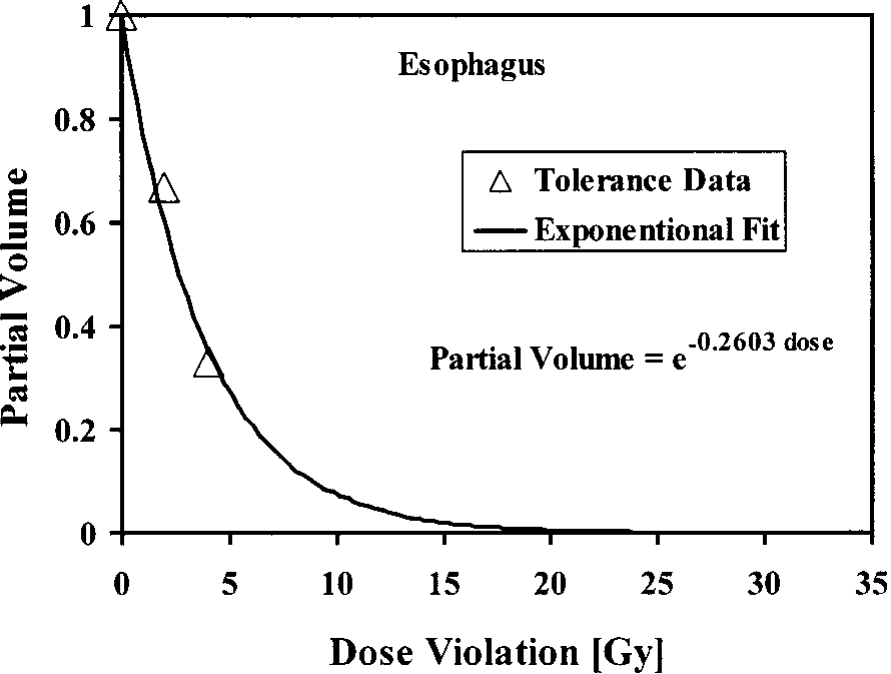
Irradiated partial volumes of esophagus as a function of dose violations for a 50% complication probability.

A range of values for $\gamma_{i}$ can be defined based on the type of normal organ and dose‐volume violations (i.e., organ‐specific dose‐volume violations) that may result in complications. In this work, the γ values for critical structures are calculated using the Emami et al. normal tissue tolerance data.^(^ ^31^ ^)^ Figure 4 shows the esophagus partial volumes (whole, 2/3, and 1/3) as a function of uniform dose violations that result in a 50% complication probability in 5 years. The figure shows, for a constant 50% complication probability, that the esophagus irradiated partial volume decreases as the amount of dose violation increases. The dose violations are calculated by subtracting the partial volume tolerance doses for whole organ, 2/3 organ, 1/3 organ $\left({\text{TD}}_{50}(1),\;{\text{TD}}_{50}(2/3),\;{\text{TD}}_{50}(1/3)\right)$ from the tolerance dose for whole organ. The esophagus γ value of 0.26 is obtained by curve‐fitting the tolerance data using an exponential function, as shown in Fig. 4. A similar procedure is used to evaluate the γ values of other critical structures.

We chose an exponential function to model the normal tissue penalty function. Normal tissue complications as a function of dose generally follow a sigmoidal distribution.^(^ ^32^ ^)^ We used the maximum penalty value in Eq. (5) associated with the worst violation for each normal tissue:

(6) $$P_{\text{NTV}}=\underset{i}{\text{min}}[P_{\text{NTV}}(V_{i},D_{i})]$$

Normal tissue subvolumes or entire volumes that are not within TDV may receive a high dose. This is especially true for IMRT, where hot spots outside the target volume occur frequently due to competing demands of target coverage and normal tissue sparing on the optimization algorithm. While ${\text{NTV}}_{\text{TD}}$ in Eq. (4) is 0 for such normal tissue, $P_{\text{NTV}}$ will penalize such a plan based on the magnitude of dose‐volume violation and thus reduces the NTSI value. For example, a lung tumor may be treated by a therapeutic dose (TD) of 60 Gy. Assume that the spinal cord is not within the TDV but within a 55‐Gy isodose volume, which is well above the tolerance limit. NTV is zero for such a plan; however, a $P_{\text{NTV}}$ value of less than 1 will penalize the plan and drive the NTSI to a lower value.

For normal tissue with a tolerance dose lower than the therapeutic dose and with only a small volume in TDV, the ${\text{NTV}}_{\text{TD}}/\text{NTV}$ value will not reflect the high risk of complications. This can be accounted for and quantified by the penalty function resulting in a low NTSI value. For example, a tumor may be treated by a therapeutic isodose volume of 70 Gy, and a normal tissue subvolume is within TDV. If the normal tissue's γ value is 0.3 and the tolerance dose for that subvolume is 50 Gy, $P_{\text{NTV}}$ have a low value, driving the NTSI down toward zero. This indicates that normal tissue sparing is not achieved despite the fact that only a small normal tissue volume lies within TDV.

For the case of normal tissue volume that is not within TDV and receiving a dose less than the tolerance dose, the penalty function can be used to penalize the NTSI if normal tissue subvolumes receive a dose more than a user‐specified dose. This can be used to differentiate between treatment plans where the target receives the same dose and normal tissues receive different doses that are below the tolerance dose but higher than a user‐specified dose.

### C. Uncomplicated target conformity index

The TCI and NTSI for all targets and critical structures are combined to derive a single figure of merit, a ${\text{TCI}}^{+}$:

(7) $${\text{TCI}}^{+}=\prod_{i=1}^{N_{T}}{\text{TCI}}_{i}\prod_{j=1}^{M_{\text{NT}}}{\text{NTSI}}_{j}$$

where $N_{T}$ is the total number of targets, and $M_{\text{NT}}$ is the total number of normal tissues. For a group of competing IMRT plans, the plan that maximizes ${\text{TCI}}^{+}$ may be considered to be the optimal plan. The ${\text{TCI}}^{+}$ provides information on the dosimetric “goodness” of a plan relative to other competing treatment plans.

A value of 1 implies an ideal plan, where target coverage with a therapeutic isodose volume is achieved with full sparing of normal tissue. A value of 0 implies the opposite with either no target coverage or irradiation of regular normal tissue volume and/or critical structures to a high untolerated dose. It is important to stress that ${\text{TCI}}^{+}$ is a comparative dosimetric index that does not provide information on the clinical outcome of the treatment plan, but is rather a quantitation of clinical merit of a treatment plan.

### D. Lung and prostate treatment plans

To demonstrate the utility of the evaluation/ranking tool, a 3D conformal treatment plan (3DCRT) and two competing IMRT plans are generated and compared for two patients with cancer of the lung and prostate. The plans are derived based on the treatment‐planning images of patients who had previously received conformal 3D external beam radiotherapy at our institution using the PLUNC (plan UNC, University of North Carolina) treatment planning software. The goal of the two IMRT plans is to better spare critical structures than the 3DCRT plan. The optimized IMRT plans are generated using the PLUNC's optimization algorithm.^(^ ^33^ ^)^ The 3DCRT plans are used clinically.

For both the lung and prostate treatment plans, the same beam energies and directions are used for each IMRT treatment site, but with different dose‐volume optimization constraints. A six‐coplanar field setup, with the gantry angles of 0°, 67°, 130°, 225°, and 292°, is used for the lung IMRT plans (IEC convention, clockwise starting from 0° at neutral position). A five‐coplanar field setup, with the gantry angles of 0°, 65°, 126°, 180°, and 245°, is used for the prostate plans. A conventional four‐field setup is used for the 3D conformal lung plan (anterior‐posterior fields with oblique off‐cord boost fields). A four‐field box is used for the 3D conformal prostate plan. All plans are generated using a 15 MV beam. A dose prescription of 70 Gy to the 95% isodose volume (i.e., TDV) is used for all lung plans. A dose prescription of 74 Gy to the 95% isodose volume is used for all prostate plans. Minimum and maximum doses of 98% and 103% relative to the prescription dose are used as the target dose‐volume constraints. As a starting point, the critical structures' dose‐volume constraints for the lung and prostate plans are defined based on the data of Emami et al.^(^ ^31^ ^)^ and Burman et al.,^(^ ^34^ ^)^ respectively.

In the ranking tool, a $D_{\text{min}}$ of 90% and a $D_{\text{max}}$ of 115%, relative to prescription dose, are used for the PTV. The lung and prostate plans are penalized if the PTV dose is higher than the prescribed dose by more than 15%, or the PTV dose is lower than the prescribed dose by more than 10%. Values of 0.04 and 0.009 are used for $\sigma_{c}$ and $\sigma_{h}$. The σ values are determined based on our in‐house clinical experience. The $\sigma_{c}$ value will result in penalties of 10%, 20%, and 30% for any target subvolume receiving a dose less than $D_{\text{min}}$ by 3 Gy, 6 Gy, and 9 Gy, respectively, and therefore will result in an unacceptable plan. The $\sigma_{h}$ value will result in penalties of 2%, 4%, and 8% for any target subvolume receiving a dose more than $D_{\text{max}}$ by 3 Gy, 6 Gy, and 9 Gy, respectively.

In the lung 3DCRT and IMRT plans, critical structure penalties are enforced by the ranking tool if the esophagus subvolumes at 30%, 20%, and 10% receive doses higher than 70 Gy, 75 Gy, and 80 Gy, respectively, and if the lung subvolumes at 30%, 20%, and 10% receive doses higher than 45 Gy, 55 Gy, and 65 Gy, respectively. The ranking tool penalized the 3DCRT and IMRT prostate plans if 50%, 30%, 20%, and 10% of the rectum volume receives doses higher than 60 Gy, 65 Gy, 70 Gy, and 75 Gy and if 50%, 30%, 20%, and 10% of the bladder volume receives doses higher than 65 Gy, 70 Gy, 75 Gy, and 80 Gy. The lung and prostate plans dose‐volume metrics used in the ranking tool are based on our institutional experience γ values of 0.26, 0.034, 0.132, and 0.072 are used for esophagus, lungs, rectum, and bladder. The values are generated using the Emami et al. critical structure tolerance data.^(^ ^31^ ^)^

The lung and prostate TCI, NTSI, and ${\text{TCI}}^{+}$ values are compared with the tumor control probability (TCP), normal tissue complication probability (NTCP), and uncomplicated tumor control probability $\left({\text{TCP}}^{+})\;[{\text{TCP}}^{+}=\;\text{TCP}(1-{\text{NTCP}}_{\text{total}})\right]$, respectively. The TCP model and parameters used in this study are based on the work of Webb and Nahum.^(^ ^35^ ^)^ In the TCP model, a value of $0.35\;{\text{Gy}}^{-1}$ is used for the mean radiosensitivity of a cell population, $\alpha_{\text{mean}}$, and a value of $0.03\;{\text{Gy}}^{-2}$ is used for β. The standard deviation $\left(\sigma_{\alpha }\right)$, or level of interpatient variability of radiosensitivity, is set to $0.08\;{\text{Gy}}^{-1}$. A constant 2 Gy fraction size is used, and a clonogenic cell density of $1\text{million}/{\text{cm}}^{3}$ is assumed. The effective doubling times for tumor clonogens $\left(T_{\text{eff}}\right)$ of 5 and 14 days are used in the lung and prostate plans, respectively. The overall elapsed time (*T*) of 42 days, over the course of radiotherapy treatment, is used. The time between the first treatment and when tumor proliferation begins (kickoff time, Tk) is set to 0. The volume dependence (*n*), NTCP versus dose slope (*m*), and the dose to reference volume leading to 50% complication $\left({\text{TD}}_{50}\right)$ parameter values, used to calculate NTCP in this study, are based on the work of Burman et al.^(^ ^32^ ^)^

## III. RESULTS AND DISCUSSION

**Figure 5:**
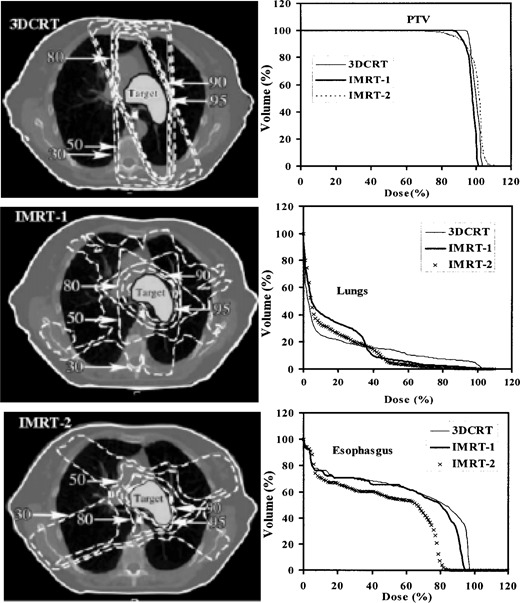
Transverse isodose distributions for a 3D conformal plan and two IMRT lung plans. The 95%, 90%, 80%, 50%, and 30% isodose lines along with the dose‐volume histograms for PTV, lungs, and esophagus are shown for all plans.

**Figure 6:**
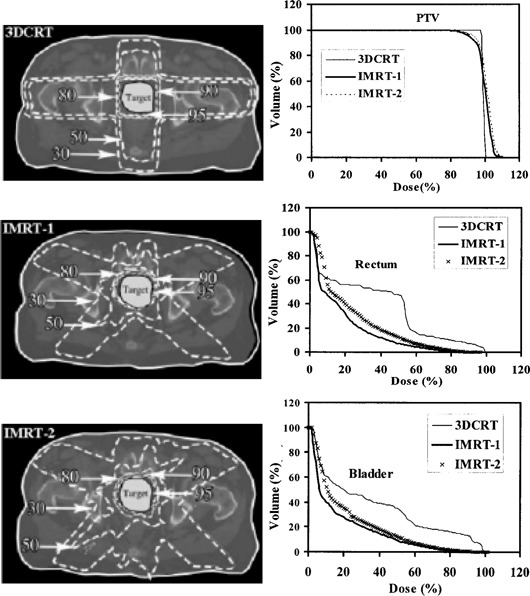
Transverse isodose distributions for a 3D conformal plan and two IMRT prostate plans. The 95%, 90%, 80%, 50%, and 30% isodose lines along with the dose‐volume histograms for PTV, rectum, and bladder are shown for all plans.

Dose distributions and dose‐volume histograms (DVHs) are calculated and compared for the lung and prostate plans. Figure 5 shows the transverse isodose distributions along with the PTV, lungs, and esophagus DVHs for the 3DCRT and IMRT lung plans. Figure 6 shows transverse isodose distributions along with the PTV, rectum, and bladder DVHs for the 3DCRT and IMRT prostate plans. For both treatment sites, we observed greater critical structure sparing in the IMRT plans at the expense of losing some target coverage, compared with the 3DCRT plan. However, what is unclear is which plan best provides optimal target coverage and optimal critical structure sparing and takes into account the tolerance doses of the critical structures. More precisely, it is not obvious how best to balance the lungs and esophagus sparing with the PTV objective of uniform dose coverage for the lung IMRT plans. Similarly, in the prostate IMRT plans, it is not obvious how best to balance the rectum and bladder sparing with the PTV objective of uniform dose coverage. In fact, despite the grouping of dose distributions and DVHs for all plans as shown in Figs. 5 and 6, we are not able to select the optimal plan based on current evaluation methods. One can make a plausible case for any of the three lung and prostate plans shown in Figs. 5 and 6. They all provide acceptable dose distributions. However, from a dosimetric point of view, one of these three plans has better dose distributions relative to the other plans. A detailed investigation of the 3D dose distributions and DVHs for all plans, which is a time‐intensive procedure, will be required for the selection of the optimal plan. However, the procedure still may not result in selecting the optimal plan if the dose‐volume effects of the target and critical structures are not incorporated in the evaluation and ranking process.

The ranking tool results of TCI, NTSI, and ${\text{TCI}}^{+}$ are shown in Table 1 for the lung plans and in Table 2 for the prostate plans, along with the TCP, (1‐NTCP), and ${\text{TCI}}^{+}$ values. The ${\text{TCI}}^{+}$ index selected the lung IMRT‐2 plan and the prostate IMRT‐2 plan as the optimal plans. The ranking tool selected IMRT‐1 and IMRT‐2 as the optimal plans for the lung and prostate, respectively. Those plans provided clinically acceptable target coverage and more sparing of critical structures resulting in the highest ${\text{TCI}}^{+}$ values of 0.80 for lung IMRT‐1 plan and 0.91 for prostate IMRT‐2 plan. TCI and NTSI values provided a single value measure of the plan's dose conformity and dose sparing. While the PTV coverage is good in the 3DCRT lung and prostate plans, the irradiation of critical structures surrounding the target resulted in lower NTSI values, compared to the IMRT plans. Despite the excellent sparing of esophagus and lungs in the lung IMRT‐2 plan as demonstrated in the NTSI lungs and esophagus values, the low TCI value (penalized for a cold spot) resulted in a low ${\text{TCI}}^{+}$ value. A similar observation is noticed for the prostate IMRT‐1 plan where high (unity) NTSI values are also observed for rectum and bladder and a low TCI value resulted in a low ${\text{TCI}}^{+}$ value.

**Table 1:** Target conformity index (TCI) and tumor control probability (TCP) for the lung target. Normal tissue‐sparing index (NTSI) and normal tissue complication probability (NTCP) for lungs and esophagus along with the uncomplicated target conformity index $\left({\text{TCI}}^{+}\right)$ and uncomplicated tumor control probability (${\text{TCI}}^{+}$) values for the lung plans. The table shows IMRT‐1 is the plan with the highest ${\text{TCI}}^{+}$ value and IMRT‐2 is the plan with the highest ${\text{TCI}}^{+}$ value.

	Target		Lungs		Esophagus		Ranking index	
Plan	TCI	TCP	NTSI	1‐NTCP	NTSI	1‐NTCP	${\text{TCI}}^{+}$	${\text{TCI}}^{+}$
3DCRT	0.978	0.950	0.947	0.998	0.503	0.578	0.466	0.548
IMRT‐1	0.814	0.935	0.992	0.995	0.997	0.727	**0.805**	0.676
IMRT‐2	0.671	0.860	0.996	1.000	1.000	0.963	0.668	**0.828**

**Table 2:** Target conformity index (TCI) and tumor control probability (TCP) for the prostate target. Normal tissue‐sparing index (NTSI) and normal tissue complication probability (NTCP) for rectum and bladder along with the uncomplicated target conformity index $\left({\text{TCI}}^{+}\right)$ and uncomplicated tumor control probability (${\text{TCI}}^{+}$) values for the prostate plans. The table shows IMRT‐2 is the plan with the highest ${\text{TCI}}^{+}$ and ${\text{TCI}}^{+}$ values.

	Target		Rectum		Bladder		Ranking index	
Plan	TCI	TCP	NTSI	1‐NTCP	NTSI	1‐NTCP	${\text{TCI}}^{+}$	${\text{TCI}}^{+}$
3DCRT	1.000	0.992	0.946	0.983	0.923	1.000	0.873	0.975
IMRT‐1	0.830	0.978	1.000	1.000	1.000	1.000	0.830	0.978
IMRT‐2	0.914	0.979	1.000	1.000	1.000	1.000	**0.914**	**0.979**

The difference in the TCP, NTCP, TCI, and NTSI values resulted in different ${\text{TCI}}^{+}$ and ${\text{TCI}}^{+}$ ranking values, as shown in Tables 1 and 2. Comparing the TCI value with the TCP value for the lung and prostate IMRT plans, we find that the tool penalized the TCI values for cold spots more severely than the TCP model, thus accounting for the user‐specified dose‐volume violations in which a severe penalty for cold spots is specified. The 3DCRT TCI and TCP values for the lung and prostate plans are higher than the IMRT values.

The lung NTSI and (1‐NTCP) values for the lung IMRT plans agree within 0.5% and approximately equal to 1, which is an indication of the excellent sparing of lungs in both plans. The lung 3DCRT NTSI value is lower than the (1‐NTCP) value. The lower 3DCRT NTSI value resulted from the fact that there is a small lung subvolume within the therapeutic dose volume. The IMRT‐1 and IMRT‐2 esophagus (1‐NTCP) values are lower than the NTSI “unity” values. The NTSI unity values reflect that there are no esophagus subvolumes within the therapeutic dose volume and/or dose‐volume violations. The esophagus 3DCRT NTSI value is lower than the (1‐NTCP) value. Furthermore, the esophagus 3DCRT NTSI value is lower than the NTSI IMRT values by 50% indicating that there are esophagus subvolumes within the therapeutic dose that have violated dose‐volume limits.

For the lung case, the ${\text{TCI}}^{+}$ value was a more reliable metric than the ${\text{TCI}}^{+}$ value. This is because the normal organs' (lung, esophagus) dose‐volume limits, defined by the physicians, were not violated and thus resulted in high NTSI values. The target TCI value in IMRT‐1 indicates better target coverage than IMRT‐2 based on the target dose uniformity window limits and the penalties associated with the dose‐volume violations. The TCP model penalized the target cold spots in IMRT‐2 less severely than TCI, and this resulted in a higher ${\text{TCI}}^{+}$ value than ${\text{TCI}}^{+}$.

Due to the excellent sparing of rectum and bladder in the IMRT plans, the NTSI and (1‐NTCP) values are unity. The 3DCRT NTSI rectum and bladder values are lower than the (1‐NTCP) unity values by 4% and 8%, respectively, demonstrating that both critical structures have small subvolumes within the therapeutic dose volume.

In addition to the ${\text{TCI}}^{+}$ and ${\text{TCI}}^{+}$ ranking results, a blind‐review study was performed in which five attending radiation oncologists at our institution were asked to review the lung and prostate plan dose distributions and then select the “optimal” plan. The purpose of this review was to investigate whether the ${\text{TCI}}^{+}$ ranking tool results agree with our physicians' preference when selecting the “optimal” treatment plan from among the rival plans. The physicians were given the dose distributions on multiple transverse, sagittal, and coronal cuts and DVHs with relevant dosimetric metrics (min dose, max dose, mean dose, volumes receiving certain doses) for the target and surrounding critical structures. No information was given to the physicians on the treatment technique (i.e., whether the plans are 3DCRT or IMRT). The physicians' ranking is shown in Table 3 for the lung and prostate cases. Four of the five physicians selected IMRT‐1 as the best plan for the lung case. The scoring of the four physicians was similar to that of the ${\text{TCI}}^{+}$ ranking tool. Physician D chose IMRT‐2 as the best plan, which was different from the ${\text{TCI}}^{+}$ ranking. Physician D was willing to accept less dose uniformity in the target coverage for additional sparing of critical structures, especially the esophagus. For the prostate plans, the five physicians chose IMRT‐2 as the best plan, which was similar to the ${\text{TCI}}^{+}$ and the ${\text{TCI}}^{+}$ ranking. The physicians indicated that the lung IMRT‐1 and prostate IMRT‐2 plans provided both “good” target coverage and “better” critical structure sparing than the other plans. The scoring results for the lung and prostate cases studied in this work show that the ranking tool is predictive of our physicians' preference and demonstrate that the tool can assist physicians in evaluating and ranking treatment plans in a clinical setting. Note that none of the physicians selected the 3DCRT plan as the optimal plan due to higher doses in the critical structures, compared to the IMRT plans.

**Table 3:** Physicians' choice of the “optimal” lung and prostate plans from among the rival treatment plans

Physician ID	Lung optimal plan	Prostate optimal plan
A	IMRT‐1	IMRT‐2
B	IMRT‐1	IMRT‐2
C	IMRT‐1	IMRT‐2
D	IMRT‐2	IMRT‐2
E	IMRT‐1	IMRT‐2

In this work, we present the ${\text{TCI}}^{+}$ model as an alternative to the ${\text{TCI}}^{+}$ model for ranking IMRT plans, especially for treatment sites where clinical data available for the TCP/NTCP models are inadequate. The TCI/NTSI indices provide information on the dosimetric properties of a plan by factoring in physician clinical experience in judging a treatment plan. This makes the ${\text{TCI}}^{+}$ customizable to an institution's experience. Specifically, the TCI and NTSI indices, unlike TCP and NTCP models, provide a method for the evaluation and ranking of dose distributions that takes into account dose‐volume tolerance data without attempting to make predictions of absolute outcome. This is an indication of the underlying differences between ${\text{TCI}}^{+}$ and ${\text{TCI}}^{+}$, viz., that ${\text{TCI}}^{+}$ is based on a biological probability model, whereas ${\text{TCI}}^{+}$ is based on clinical judgment. Thus, for example, the constituent terms of ${\text{TCI}}^{+}$ (TCP and NTCP) continuously penalize tumor and normal tissue, whereas the constituent terms of ${\text{TCI}}^{+}$ (TCI and NTSI) only apply penalties when doses violate set limits. It should be pointed out that Langer et al.^(^ ^36^ ^)^ reported that the ${\text{TCI}}^{+}$ score function should not be used to draw conclusions on treatment techniques without statements of errors in the TCP and NTCP values. Moreover, the ${\text{TCI}}^{+}$ approach weights a complication equal to a tumor relapse, which is certainly not clinically realistic. A number of studies suggested the use of weighting coefficients in the ${\text{TCI}}^{+}$ score function to allow for differences in tissue importance and the use of critical elements architecture for calculating NTCPs.^(^ ^37^ ^,^ ^38^ ^)^

For treatment plans with dose‐volume violations, the accuracy of the TCI and NTSI values depends on the accuracy of the clinical tolerance data that are used to generate the parameter values for the penalty functions. To use the model clinically, one may initially fit the parameters of the ${\text{TCI}}^{+}$ model using in‐house clinical experience (i.e., the physicians define dose‐volume limits and the penalties associated with the dose‐volume violations). The best application of the ranking tool will be obtained when treatment planners compare the indices with their own clinical experience. If the indices provide information that matches clinical experience, this will suggest that the parameter values are appropriate. However, if the calculated indices repeatedly differ from their own clinical experience, new parameter values for the penalty functions can be calculated based on in‐house experience.

Treatment planners routinely evaluate different IMRT plans, that is, with different beam arrangements and modulation/weighting before settling on a final plan. The process of deciding on a final plan takes into consideration numerous dosimetric factors and hence is suboptimal when manually conducted. The ${\text{TCI}}^{+}$ tool provides an automated way for evaluating and choosing between different competing plans. The ranking tool can be used for the evaluation of conventional dose distributions and is not limited to 3D conformal and/or IMRT plans. The tool was applied to 3D conformal and IMRT plans in order to stress the importance of using dose‐volume metrics for ranking dosimetrically guided dose distributions. While the TCI and NTSI indices presented in this work are used to evaluate and rank IMRT treatment plans, they can also be used as objective functions in optimization algorithms.

The subject of the sensitivity of parameter selection in the ${\text{TCI}}^{+}$ ranking tool is not dealt with in this work. This work is presently under development, and we plan to report the results in a future article.

## IV. CONCLUSIONS

A tool to compare competing IMRT treatment plans, based on dose‐volume indices, is developed and presented. The tool uses physical parameters to represent complex dose distributions as a single value of merit. Results show that a clinically relevant index can be used to select the optimal plan from among rival treatment plans and is more appropriate than traditional methods for evaluating/ranking IMRT dose distributions. The tool is flexible and allows for user/organ‐specific considerations.

## ACKNOWLEDGMENT

This work is partially supported by NIH grant 69579 and Varian Medical Systems.
